# Supplementary material for: Complexome profiling of the Chlamydomonas psb28 mutant reveals TEF5 as an early PSII assembly factor
Source: Plant Cell. 2025 Mar 17;37(6):koaf055. doi: 10.1093/plcell/koaf055 (PMC12164590; doi:10.1093/plcell/koaf055)
Supplement: koaf055_Supplementary_Data [file koaf055_supplementary_data.zip › psb28_interactive.zip!/plots/intensity/qualityPlots/peptides/NUOP4_Cre08.g378550.t1.2.html]

### Cre08.g378550.t1.2

Trivial name: NUOP4  
SeqCoverage [median]: 48.70  
Mass: 8.72 kDa  
  
 

| Sequence | MissedCleavage | Mass [Da] | wtIntensity | muIntensity | Score |
| --- | --- | --- | --- | --- | --- |
| LNYNFEPMGMGSLFIR | 0 | 1887.90 | 1177.19 | 0.00 | 43.79 |
| VDTSLDHVFGDNSNK | 0 | 1646.75 | 17210.72 | 10326.74 | 121.31 |
| YTSFLDK | 0 | 872.43 | 2344.89 | 1452.17 | 79.12 |
|  |  |  | 20732.8 | 11778.9 |  |
